# Supplementary material for: Are carbon emissions trading and green financial instruments synergistic? -Comprehensive quantitative research based on content analysis
Source: PLoS One. 2024 Mar 7;19(3):e0298601. doi: 10.1371/journal.pone.0298601 (PMC10919601; doi:10.1371/journal.pone.0298601)
Supplement: S1 Data — (ZIP) [file pone.0298601.s001.zip › Data/地级市Z_F工作报告.zip!/╣π╢1⁄2/╘╞╕í╩╨/└┤╫╘╣1⁄2╓┌║┼í╛┬φ┐╦╩2╛▌═°í┐_2016.docx]

各位代表：

我代表市政府向大会作政府工作报告，请予审议，并请政协各位委员和其他列席人员提出意见。

2015年及“十二五”时期工作回顾

2015年是“十二五”规划收官之年。一年来，市政府在省委、省政府和市委的正确领导下，在市人大及其常委会和市政协的监督支持下，深入贯彻落实党的十八大、十八届三中、四中、五中全会和习近平总书记系列重要讲话精神，紧紧围绕全面建设现代生态城市目标，牢牢把握“跨越赶超、科学崛起”主题，坚持生态立市、产业兴市、特色美市、改革活市、依法治市，狠抓“招商、建城、改革”，促进经济增长稳中有进、稳中提质，社会事业取得新成就，保证了“十二五”规划主要目标任务的基本完成。预计全年实现地区生产总值710.07亿元，比上年增长8.5%；地方一般公共预算收入58.7亿元，增长9.3%；规模以上工业增加值289.23亿元，增长9.5%；固定资产投资794.15亿元，增长17.6%；社会消费品零售总额299.71亿元，增长11.6%；城镇、农村常住居民人均可支配收入分别达20172元、11952元，均增长8%；居民消费价格上涨1.2%；完成省重点建设项目年度计划投资112.9%。

一、加快发展现代产业，调结构促转型取得显著成效

（一）新产业培育亮点纷呈。坚持在扩容增量中综合施策调结构，三次产业结构为21.1 :43.7 :35.2。着力发展“四新一特”产业。云计算和信息服务产业发展取得实质性突破，编制实施《云计算及信息服务产业发展规划》和《“智慧云浮”建设方案》，“一中心三平台”建设有序推进。成功引进华为公司参与“云谷”建设，中国网库、神州数码、亿赞普集团、华唐中科教育集团等企业相继落户。与佛山市联手共建先进装备制造业合作示范基地，装备制造业规模以上企业发展到31家。市健康医药产业园和罗定中药提取产业基地建设全面加快，广东一力罗定制药GMP技改项目试产，生物医药规模以上企业发展到11家。积极发展循环经济和新能源汽车、新材料产业，罗定成为国家循环经济示范城市（县）建设地区，郁南虎头节能电源等项目建设加快。

（二）现代农业稳健发展。实现农林牧渔业总产值238.78亿元，增长4.2%。全国农村改革试验区建设扎实推进。我市成为全国四个国家农产品质量安全市创建试点单位之一。农业综合开发、现代特色农业产业示范基地建设全面加快，建成高标准基本农田32.14万亩。温氏禽畜、罗定稻米、新兴茶叶、郁南无核黄皮等一批农业龙头企业标准化生产或加工基地不断发展壮大，农业产业化组织发展到2497个。新增有机食品认证3个、无公害农产品认证18个、省名牌产品（农业类）5个，入选省名特优新农产品目录库25个。广东温氏食品集团股份有限公司在深交所创业板成功整体上市；130家企业在不同的区域股权交易市场挂牌融资。

（三）现代服务业加快发展。实现现代服务业增加值125.3亿元，增长9.4%。成功举办了第十二届中国（云浮）国际石材科技展览会暨第六届中国（云浮）石文化节、广东云浮·罗定稻米节。大力建设“全国智慧旅游试点市”，以广东禅文化创意产业园为代表的健康养生旅游产业加快推进。我市被评为“中国特色养生休闲旅游最佳目的地”。全年接待游客2062.59万人次，增长9.9% ，实现社会旅游总收入214.59亿元，增长9.02%。金融服务对地方经济发展支持力度加大，12月末金融机构各项存款、贷款余额分别为915.88亿元、598.05亿元，比年初分别增长10.84%和12.14%。

二、全面推进载体平台建设，发展后劲不断增强

（一）扩容提质建设全面加快。云浮新区核心区——西江新城建设稳步推进。南广铁路交通枢纽、中央商务区、北湖启动区等项目有序推进。云安区的商贸金融、文化教育、医疗卫生、居住、行政服务等功能加快向西江新城集中。市“三合一”行政服务中心投入使用。市中心城区公共服务设施进一步完善，河滨西路、金丰路、金山路（北段）等3条市政道路全线贯通。罗定、新兴、郁南城区扩容提质初显成效，城区面貌变化喜人。全市完成“三旧”改造项目17个，改造面积743亩。罗定市、郁南县平台镇和市“一张蓝图”工程分别被省住建厅列为省新型城镇化“2511”综合试点县、镇和专项项目。

（二）工业园区建设全面加快。重点工业园区累计入园项目318个，投资总额523.36亿元，实现规模以上工业总产值338亿元，规模以上工业增加值88亿元，占全市比重31%、30.8%。以佛山（云浮）产业转移工业园为依托，成功申报了思劳片区、云安片区、罗定片区、郁南片区等四个产业集聚地，并采取“一主带多副”的模式，纳入省产业转移园统一管理，实现全市园区联动发展。华润西江发电厂项目获省发改委核准，海尔集团等一批知名企业落户发展。德磁科技、新创环保、国琳铝业等一批优质项目竣工投产。顺德（新兴新成）产业转移工业园实现规模以上工业增加值11.44亿元，增长36.7%。

（三）合作平台建设全面加快。佛山方面帮扶力度加大，财政帮扶资金增加到16亿元，建立了16亿元的股权投资基金。引进佛山市企业项目（含签约项目）93个，计划总投资348亿元。思劳片区在园区开发和项目建设上取得新突破，飞驰氢能源整装客车等一批大项目落地建设，益诺欧超滤环保膜、氢燃料电池模块生产等项目即将建成投产。两市各区县深入对接，共建合作园区取得新成效。全面启动教育及卫生专业人才千人互派计划，互派人员200名。积极推进粤桂黔三地交流合作，省政府同意设立粤桂黔高铁经济带合作试验区广东园云浮分园。顺利举办“两广十市”区域旅游合作联席会议。举办经贸洽谈会、招商引资洽谈会、招商项目推介会等30余场次，新引进投资项目225个、市外资金439亿元。依托广州南沙港，云浮（罗定）海关率先启动全省首个“无水港”通关试点，通关效率大幅提高。

（四）服务平台建设全面加快。大力搭建政银企服务平台，举办银企融资对接会及培训会10场，一大批企业融资难问题得到缓解。金融服务体系建设不断加快和完善，引进或新设各类金融机构10个，其中已投入运营6个。创新金融服务，设立“四平台四基金”。创新驱动发展战略深入实施，以“1+10”体系支撑科技创新力度加大。积极实施“互联网+”行动计划，云浮高新区科技企业孵化器建设扎实推进，云浮“数字人社”在全省推广。新建省、市级企业工程技术研究中心(实验室) 4个，签订科技合作协议29项，申请专利876件，增长30.2%。取得《不锈钢制品绿色制造工艺及自动化生产线共性技术研究》等重大科技成果41项，获得全省科技进步奖4项，其中一等奖2项。新增惠沄钛业股份有限公司等国家级高新技术企业5家，有15家企业进入省级高新技术企业培育库。实施科技成果转化项目35个，实现利润1.36亿元，增长15.2%；规模以上工业实现高技术产业总产值 82.6亿元，增长23.16%。质量强市工作加快推进，食品药品检验检测中心建成并通过验收，新增省名牌产品（工业类）10个。

三、全面推进城乡统筹发展，城乡区域互促共进

（一）大交通体系初步形成。投入建设资金73.88亿元，为历年之最。江罗高速高村至新兴段、广梧高速高村立交建成通车；罗阳、汕湛高速云浮段等重点交通设施建设进度加快，高恩高速公路动工建设，罗定至德庆高速公路北段纳入省高速公路中远期规划，高速铁路运营里程、高速公路通车总里程分别达到106公里、209公里。城乡交通同步推进，国省干线联网畅通，新城快线一期建成通车。S113线、S369线等共195公里升级为国道。新建农村公路420公里，改造提升汽车客运站3个。完成罗岑铁路股权回收工作。西江航道升级、南江口码头扩能等项目加快推进。罗定机场的现代通用航空产业加快培育发展，珠三角新干线机场项目初步列入省“十三五”规划。

（二）良好生态环境体系基本建成。深入开展新一轮绿化广东大行动，完成造林面积22.81万亩，森林覆盖率69.5%；绿化村庄133条。新增国家级湿地公园（郁南大河）1 个。新农村建设取得新成效，建设生态文明村3308条，建成宜居城镇5个、宜居村庄 77条、宜居社区5个，新增广东名镇2个、名村27条、全国“一村一品”示范村1条。强力推进以治理违法违规企业、西江岸线码头、中小河流、车辆超限超载、农村生活垃圾和污水为重点的“五治”工作，成效明显。整治违法违规企业1670多家、码头19个。全市8家硫化工企业涉重金属、废水均实现零排放。扎实推进村村通自来水、小型农田水利、中小型灌区改造等工程项目建设，除险加固中小型水库12座，改造农田水利渠系510公里。狠抓重点流域水环境综合整治和饮用水源保护，主要江河湖库水质保持良好，获得全省珠江综合整治考核山区城市第一名、全省污水处理设施考核优秀等次。全面启动14个中心镇及150个农村生活污水处理示范点建设，实现城镇生活污水集中处理率75.1%。中心城区城市生活垃圾无害化处理率100%，农村生活垃圾无害化处理率92.92%。在2015年度省农村生活垃圾收运处理工作第三方评估中，居粤东西北地区第一位。淘汰黄标车及老旧车辆1.7万辆。顺利完成省下达节能目标和主要污染物减排任务，单位生产总值能耗下降2.5%；空气质量保持在国家二级标准以上，是全省唯一的市区空气质量优良率全面达标内陆城市。耕地保护工作成效显著，连续15年实现耕地占补平衡。

四、全面推进社会事业建设，民生保障不断加强

全年民生类支出125.12亿元，占全市地方一般公共预算支出的76.45%，十件民生实事较好完成。

（一）创业就业和社会保障工作成绩喜人。大众创业、万众创新工程深入实施，扶持创业1231人，新增城镇就业3.4万人，城镇登记失业率2.47%。新增转移就业3.24万人，组织劳动力技能晋升培训3.8万人。省人事考试云浮基地建成使用。城乡居民养老保险及医疗保险实现全覆盖，大病医疗保险制度全面实施，社会保险经办管理实现“一卡通”，医疗保险实现省内就医异地即时结算。职工社会保险“五险种”累计参保119.2万人次。城乡低保对象实现应保尽保。为16632名重度残疾人发放护理补贴2993.76万元,为13705名残疾人发放生活津贴1569万元。完成棚户区改造450户（套），开工建设各类保障性住房2114套（户），建成保障性安居工程项目10个1060套，向农民工提供公共租赁住房535套，完成农村危旧房改造4276户。圆满完成第二轮扶贫开发“双到”工作各项任务，减少农村贫困人口12.58万人，在省考核验收中获优秀等级。

（二）教育强市工作稳步推进。教育强市创建工程总体完成，实现了教育强县全覆盖，省教育强镇覆盖率达到88.9%，公办义务教育标准化学校达到100%。现代职业教育改革加快推进，现代远程开放教育和民办教育健康发展，云浮恒大高级中学加快建设，云浮市伊顿实验学校新校区建成使用。顺利通过省二类城市语言文字工作评估达标验收。

（三）文化体育工作协调并进。建成市级公共文化服务示范区和示范项目各1个，建成村落历史文化展示馆和非物质文化遗产展示馆一大批。文化申遗工作取得新进展，禅宗文化、石艺文化、南江文化等文化品牌影响力持续提升，“非物质文化遗产（优秀传统文化）进校园”被评为省2015年度文化志愿服务优秀项目。全民健身运动蓬勃发展，建成镇级农民体育健身广场9个，实现镇级“农民体育健身广场”全覆盖。第二届“市长杯”足球联赛成功举办，与上届比规模更大、影响更广。参加省第十四届运动会收获4金4银7铜，参加省第八届残运会收获13金2银1铜。

（四）卫生计生工作扎实惠民。县级公立医院综合改革覆盖率100%，有7家县级公立医院全面取消药品加成并调整医疗服务价格。医疗卫生服务水平显著提升，城乡居民规范化电子健康档案建档率92.7%；登革热和人感染H7N9禽流感等传染病有效防控，无重大传染病疫情暴发流行和突发公共卫生事件发生。人口与计划生育工作完成年度目标管理任务，常住人口自然增长率7.26‰，户籍人口政策生育率92.13%，控制在省下达指标内。新兴县、郁南县成功创建省卫生县城。

（五）平安创建工作成效突出。“平安云浮”建设深入推进，严厉打击各类违法犯罪活动，未发生重大影响的恶性刑事案件和治安案件，公众安全感和满意度名列全省前列。“无毒城市”创建活动深入开展，禁毒涉毒专项整治工作成效显著。安全生产责任体系不断完善，企业主体责任有效落实，四项主要控制指标全面下降。工商、金融监管不断加强，打击走私的基础保障和综合治理工作不断加强并取得明显成效，社会秩序和市场秩序得到规范；食品药品、农产品安全监管继续加强，没有发生大的食品药品安全事件。气象建设进一步加快，建成市级和罗定、新兴、郁南3个县级突发事件预警信息发布平台和63个镇（街）气象服务站。积极贯彻依法治军、从严治军方针，军分区部队和民兵预备役建设稳步发展，国防动员、国防教育和国防后备力量建设扎实推进。

五、全面推进法治政府建设，改革活力不断激发

（一）依法行政深入推进。全面完成“六五”普法任务。科学民主决策水平不断提高，市政府常务会议工作规程和重大决策专家咨询评估制度严格执行。行政权力监督不断加强，行政执法行为不断规范，行政审批制度改革不断深化，市级全年调整压减审批事项304项，取消非行政许可事项类别。企业投资项目负面清单管理试点工作扎实推进，市级共梳理出市直部门权责事项6703项。市公共资源交易中心正式运行。认真开展政府投资项目招投标秩序和重点工程施工环境专项治理。政府信息、决策、执行、管理、服务和结果公开工作不断深化。认真执行市人大及其常委会各项决议并报告工作，主动向政协通报情况，广泛听取民主党派、工商联、无党派人士和各人民团体意见，办理人大代表建议88件、政协委员提案132件，办结率、满意率100%。有效解决行政复议案件39宗、行政诉讼案件19宗。

（二）综合改革加快推进。“三证合一、一照一码”、企业注册资本实缴改认缴、年检改年报、信用信息公示等商事制度改革全面实施。不动产登记职责整合全面完成，启动不动产登记工作。落实省政府投资项目核准目录，积极推动投融资体制改革，鼓励社会资本投资重大项目。农村金融创新步伐不断加快，“三农”融资难问题得到缓解。“营改增”试点、财政绩效、预算管理和政府性债务管理改革加快实施，税收分享体制改革机制初步建立。城乡一体化户籍改革方案正式出台，质监、工商部门行政管理体制改革和公务用车制度改革、市城区阶梯气价改革如期完成。就业和社会保障、人事人才、教育、科技、文化、医疗卫生等领域改革取得明显实效。民营经济发展体制机制不断完善，实现民营经济增加值493.9亿元，增长8%。

（三）服务型政府建设有效推进。网格化社会管理服务体系不断完善，社区管理服务能力全面提升。法律进社区、进农村活动扎实开展，服务群众体系不断健全。创新信访工作，依法调处化解矛盾纠纷，一大批积案难案得到妥善化解。严格推进社会稳定风险评估工作，条块结合、纵横联动、覆盖全域的评估体系基本形成。扎实开展“三严三实”专题教育，政府施政能力和执行力大大提升。严格执行中央“八项规定”、国务院“约法三章”、省委省政府“十项规定”和市委有关规定，“三公”经费和会议费支出同比下降15.83%，所节约资金均投向民生事业。认真落实政府部门党风廉政建设责任制，严格履行“一岗双责”。严厉惩治腐败行为，政府系统作风建设和廉政建设进一步加强。

此外，民族宗教、外事侨务、港澳事务、台务、人民防空、统计、地方志、消防、档案、保密、侨联、文联、社科联、妇儿、工商联、红十字会、贸促会、代建服务以及驻市外机构等工作也取得了新成绩。

各位代表，我市“十二五”规划目标基本实现。过去的五年，是我市全面加快发展的五年、夯实基础的五年、继往开来的五年：

——五年来，综合实力稳步增强。全市地区生产总值年均增长11.7%；地方一般公共预算收入年均增长20.5％；规模以上工业增加值年均增长22.5%；社会消费品零售总额年均增长16.9％；固定资产投资额年均增长20.6%；城镇居民人均可支配收入年均增长10.7%；农村常住居民人均可支配收入年均增长12.8%。

——五年来，交通设施日臻完善。完成资金投入307.08亿元，是“十一五”时期的1.76倍。南广铁路、云罗高速、江罗高速高村至新兴段、河杨公路、新城快线等一批重点工程项目相继建成，国省道改造升级和迎国检任务顺利完成，水陆铁空一批重点交通项目得到谋划建设，顺利实现“县县通铁路、县县通高速公路”目标任务。

——五年来，工业基础持续夯实。累计引进企业项目1059个，投资总额达 2107.32 亿元，其中亿元以上项目 256 个。外贸进出口累计 81.37亿美元，年均增长7.2%，是“十一五”时期1.51倍。工业加速转型升级。淘汰落后和化解过剩产能企业13家。产业投资持续增加，完成工业投资1328亿元。

——五年来，城乡环境持续优化。市中心城区和各县（市、区）城区品位显著提升，高起点规划建设的西江新城初见成效。城乡环境综合治理成效显著，镇村形象更加靓丽。防灾减灾能力显著增强，森林覆盖率持续提高，水环境保持良好，空气质量持续改善，为建设现代生态城市打下了坚实基础。

——五年来，社会管理全面提升。建立健全综治维稳、消防、安全生产、应急管理长效机制，坚持开展民间纠纷调处、劳动争议调解、市镇领导接访等行动，各类事故指标、刑事案件和信访数量稳步下降，协同共治体系建设深入推进，社会治理能力全面提升，群众安全感明显增强。

——五年来，民生事业全面发展。民生类支出累计投入417亿元。公共文化服务体系建设、教育强县、义务教育均衡县和社区医疗卫生服务实现了全覆盖，居民基本养老保险和基本医疗保险实现了城乡统筹，人口和计生事业稳步发展。医疗卫生资源全面整合，成功创建4家“三甲”医院，并在全省地级市率先创建成为“全国基层（社区和农村）中医药工作先进单位”。城市居民和农民收入稳步增长，最低工资和最低生活保障标准逐步提高。城乡公共服务日臻完善，市民综合素质不断提升。云浮进入国家卫生城市和广东省文明城市行列。

——五年来,改革创新砥砺奋进。在行政审批、试验区建设、普惠金融发展等方面取得令人瞩目成就。2013年、2014年全省促进粤东西北地区振兴发展行政审批制度改革评估考核被评为优秀等次；我市全国农村改革试验区建设经验得到国家肯定并在全国推广；国家农产品质量安全市创建试点工作扎实推进，在全省率先实现农产品质量安全监管公共服务机构100%覆盖到镇、延伸到村；农村普惠型金融体系不断完善，郁南经验在粤东西北地区农村金融工作现场会上推广。

取得的成就，令人振奋；积累的经验，弥足珍贵。我们深切体会到：**一是**必须坚持把发展作为全面建设现代生态城市的第一要务，千方百计推动经济跨越式增长。**二是**必须坚持把工业作为经济发展的第一重心，千方百计推进新型工业化进程。**三是**必须坚持把“招商、建城、改革”作为主要任务，千方百计增强发展活力。**四是**必须坚持把增进民生福祉作为政府施政的第一目标，千方百计提高群众生活指数和幸福指数。**五是**必须坚持把城乡统筹作为加快发展的重要理念，千方百计营造宜商宜业宜居的发展环境。

云浮已经站在了一个新的历史起点。这是省委、省政府和市委正确领导、科学决策的结果，是全市人民齐心协力、不懈奋斗的结果。在此，我代表市人民政府，向全市人民，向人大代表、政协委员，向各民主党派、工商联、无党派人士和人民团体，向驻云浮部队、武警官兵、政法干警和民兵预备役人员，向所有关心支持云浮的同志们、朋友们，致以崇高的敬意和衷心的感谢!

各位代表！总结五年来的工作，我们也清醒地看到还存在一些亟待解决的困难和问题：**一是**部分经济指标增速有回落趋势，投资高速增长难以维持，出口困难加剧，稳增长难度加大。**二是**产业结构有待调整优化，一些传统产业做大做强步速不快，新落地的重大新兴产业项目不多。**三是**生产要素成本上升，中小企业融资难问题依然突出，生产经营困难增多。**四是**一些改革举措跟不上经济社会发展形势，妨碍发展的体制机制障碍亟待破除。**五是**群众对医疗、教育、食品安全、城乡建设等还有不够满意的地方，基础设施、公共服务、民生改善、扶贫攻坚等任务还十分艰巨。**六是**在政府工作和自身建设方面，有些决策部署和工作措施还没有落到实处，依法行政还需加强，一些领域腐败问题仍然存在。我们将本着对党和人民高度负责的态度，直面应对，勇于担当，认真解决。

“十三五”时期奋斗目标和主要任务

“十三五”是全面建成小康社会的决胜阶段。做好“十三五”时期的各项工作，意义重大，影响深远。综观国内外形势，我市发展仍处于可以大有作为的重要战略机遇期，同时也面临诸多矛盾叠加，风险增多的严峻挑战。一方面，国家布局“四个全面”和实施“一带一路”战略，推进实施珠江-西江经济带发展规划；省委省政府大力促进粤东西北地区振兴发展，构建“广佛肇清云韶”经济圈，规划建设粤桂黔高铁经济带合作试验区广东园云浮分园，佛山市对口帮扶工作深入推进，这些都是我市加快发展的有利因素。另一方面，我们也要看到，云浮发展底子薄、基础弱、总量小，要保持经济中高速增长，提高质量效益的压力和难度还很大。我们务必坚定信心，积极作为，奋力后发赶超，努力完成“十三五”时期各项目标任务。

“十三五”时期市政府工作的指导思想是：深入贯彻落实党的十八大、十八届三中、四中、五中全会和习近平总书记系列重要讲话精神，按照“四个全面”战略布局，坚持创新、协调、绿色、开放、共享的发展理念，统筹推进经济建设、政治建设、文化建设、社会建设和生态文明建设，把握“跨越赶超、科学崛起”主题，按照“生态立市、产业兴市、特色美市、改革活市、依法治市”要求，以提质增效升级为发展导向，以创新驱动发展为转型动力，不断提升产业支撑力、城市竞争力和营商环境承载力，不断增进民生福祉，全面建设现代生态城市，全面建成小康社会。

——我们要坚定不移提升云浮经济总量和城乡居民收入水平。今后五年，我市经济增长预期目标是：地区生产总值年均增长11%。到2018年，地区生产总值和城乡居民人均收入比2010年翻一番，与全省同步建成小康社会。到2020年，全市地区生产总值约1200亿元，城乡居民人均收入达到全国同期平均水平。

——我们要坚定不移建设现代生态城市。到2020年，城镇生活污水处理率达到80%，城市生活垃圾无害化处理率达到100%，耕地保有量保持12万公顷，森林覆盖率达到70%，中心城区人均公园绿地面积达8.4平方米。建设一批森林公园、湿地公园、城市山水景观等项目，建成森林公园118处，其中国家级森林公园1处、省级森林公园4处。力争全市集中式饮用水源水质优良率100%，空气优良率达到90%以上。切实提升云浮新区生态优势，增强生态环境吸引力。

——我们要坚定不移推进立体交通网络建设。到2020年，计划投资409.68亿元。推进辖区汕湛、怀阳、云茂等高速公路建设，完成高速公路建设223公里，高速公路通车里程突破430公里。推动广茂铁路、春罗铁路升级改造，尽快启动罗岑铁路和西江大桥建设。加快推动广茂铁路云浮站至云浮新港线动工，联手谋划湛江-肇庆-广州-河源-梅州（云浮段）的铁路客运专线通道，铁路通车里程新增46公里，总里程达到302公里。力争珠三角新干线机场落户佛山与云浮交界地带，推进罗定机场升级改造为支线机场。加快完成西江航道扩能改造，统筹规划建设沿江港口码头。强化市域交通无缝对接，抓好新城快线二期、东部快线等城市组团交通干线建设，加快推进和落实国省道、县乡公路升级以及一批农村公路硬底化改造计划，新改建国省道和农村公路里程2500公里。积极谋划和推动高速公路经济带、高速铁路经济带、临港物流经济带建设。

——我们要坚定不移建设和谐幸福家园。到2020年，文明村镇覆盖面达100%。大力保障和改善民生，让发展成果更好地惠及人民群众。到2018年，实现基本公共服务均等化，完成全面建成小康社会的各项任务。城乡居民基本养老保险和基本医疗保险覆盖率达98%以上，全市住房保障总体达到全省中上水平，人口出生率和自然增长率控制在省下达指标以内。城镇新增就业10万人，城镇登记失业率在4%以内。高等教育毛入学率达到40%，创建教育现代化先进县覆盖率100%。开展精准扶贫、精准脱贫，全力以赴确保完成5万多户12万多人脱贫目标任务。

2016年工作安排

2016年是实施“十三五”规划开局之年，也是全面深化改革关键之年。综合考虑各方面因素，今年的主要预期目标是：地区生产总值增长10%；人均地区生产总值增长9.7%；地方一般公共预算收入增长10%；规模以上工业增加值增长13.5%；固定资产投资总额增长12%；社会消费品零售总额增长12%；居民消费价格涨幅在1.8%左右；外贸进出口总额增长3%；城乡居民人均可支配收入增长高于同期GDP增速；完成省下达的节能减排任务。安排83项重点建设项目，计划总投资1231.84亿元，年度计划投资193.08亿元。

今年经济社会发展任务十分繁重。我们将深入贯彻落实习近平总书记系列重要讲话和中央经济工作会议、省委十一届六次全会和市委五届七次、八次全会精神，围绕结构深度调整、做大做强实体经济这一主线，主动把握经济发展新常态，以新理念引领云浮新发展，在“招商、建城、改革”上再下功夫，大力抓好供给侧结构调整，切实去产能、去库存、去杠杆、降成本、补短板，做优增量、调整存量、主动减量，努力提高经济发展质量和效益，保持社会和谐稳定，为实施“十三五”规划和全面建成小康社会开好局。重点抓好六大方面工作：

一、更加注重转型升级，着力推进经济提质增效

（一）大力推动新兴产业发展。突出“四新一特”引领。**加快发展云计算及信息服务产业。**完善《云计算及信息服务产业发展规划》和“云谷”规划设计，启动云计算数据中心建设。大力实施“智慧云浮”工程，积极引导云服务范围扩充，推动云计算大数据广泛应用。加快新富云广东电商创业孵化示范基地等电商项目建设，鼓励电商产业发展壮大。积极实施电商广货网上行，扩大城乡消费。**加快发展先进制造业。**扎实推进氢能源产业基地、精密机械装备制造及海洋装备配套项目建设，重点发展汽车零配件、环保设备、石材装备、精密机械、智能装备、海洋装备配套、农牧机械制造等先进装备制造产业，打造区域性合作示范基地。**加快发展生物医药产业。**以市健康医药产业园、罗定中药提取产业基地为支撑，加快好依护、朗圣医药、泰康制药、德澳药业、新金山生物科技等项目建设，形成生物医药产业集群发展。**加快发展健康养生旅游产业。**以禅文化创意产业园、兰寨南江文化创意基地、陈璘文化创意产业园建设为支撑，整合南江文化、石艺文化、农业观光、健康养生等领域资源，发展文化、养生、养心等产业，打造文化交流高地和健康产业、休闲养生目的地，支持新兴县创建“国家养生旅游示范基地”。**加快发展现代特色农业。**推进江罗（禅宗文化）生态农业产业带、广梧—西江南岸（美丽田园）休闲农业观光带建设，统筹跨区域农业合作。推动罗定稻香园等一批农业企业在新三板上市。积极探索“互联网+”合作模式。推动“都市农业、现代园艺，美丽田园、现代粮仓，禅意农耕、现代农牧，岭南果园、现代桑田”等农业功能分区，促进花卉、园艺、水稻、油茶、蚕桑、水果等产业转型升级。

（二）大力改造提升传统产业。鼓励传统产业深入推进新一轮技术改造，加快扩产增效、设备更新和智能化、绿色化步伐。**推动石材产业升级做强。**继续办好石展会和石文化节，引导石材产业向文化创意、工程设计、精细化发展；抓紧建设国家石材数据库和省质量监督石材加工机械检验站（云浮），制定一批具有竞争力的国家（国际）标准。**推动水泥产业向生态环保型发展。**充分利用本地适用的工业尾（废）料作为混合材料资源，鼓励下游产品延伸和发展，通过资源再生、循环利用，有效降低水泥生产成本和降低排放。**推动不锈钢制品产业创新发展。**鼓励开展产业共性技术和关键技术攻关，构建标准化技术和信息服务支撑体系、企业间分工协作体系，打造区域品牌。支持凌丰集团创建中国现代餐厨具转型升级产业基地。**推动硫化工产业延伸产业链。**深度开发硫化工产品，提高综合利用水平。同时，支持云浮发电厂、粤泷发电厂加快技术改造，提高效益。支持电子出口企业扩能增产。支持电池产业技术升级，推动产品向动力型电池转型。

（三）大力推动载体平台建设。积极探索多种投资和合作建设模式，加快园区基础设施和公共服务配套建设，强化各重点园区的集聚效应。全力加快思劳片区在建项目和路网等配套设施建设，争取一批重点项目建成投产，同时筹建氢能产业及新材料发展研究院；加快云安片区绿色日化产业集聚区、罗定片区海尔云浮（罗定）创业园、郁南片区新型化工建材项目建设；推动顺德（新兴新成）产业转移工业园扩容提质和产业集聚取得新成效。力争年内全市重点产业园区实现规模以上工业总产值突破400亿元、规模以上工业增加值突破100亿元，占全市比重35%以上。完善“智慧招商”信息化平台建设，推进招商引资深度开展。加强与国内外驻穗机构和行业商协会的沟通合作，积极拓展对外交流。依靠佛山对口帮扶力量，深化产业合作，力争引入一批大项目、好项目。健全大项目跟踪台账制度，提高项目履约率和落地率。继续开展金融风险专项整治，预防和化解金融危机。年内择优选取20家潜力企业进入上市后备企业库。加大培育力度，推动4至6家企业上市，促进企业利用资本市场做大做强。推动“四平台四基金”高效运作，积极为企业融资提供支持。

（四）大力推动科技创业创新。构建政府、企业和人才“三位一体”的创新体系，推动科技、金融、人才、产业融合发展。增强产学研的针对性，提高服务企业和孵化科技成果能力。积极实施高新技术企业培育计划，争取年内3到5家获高新技术企业认定。以技术改造为抓手，推进传统产业开展“机器换人”应用。加快推进产品创新、工艺创新、产业组织创新、管理创新和商业模式创新，引导不同产业进行差异化定位。鼓励建立新型研发机构和产业技术创新战略联盟，创建协同创新、联动创新机制。深入实施质量强市战略，打造“云浮创造”、“云浮质量”、“云浮品牌”。

二、更加注重环境建设，着力提升现代生态城市水平

（一）加快立体大交通建设。年内计划投入资金79.02亿元。全面推进水陆铁空交通建设，构筑以“两纵四横”为一体的六廊综合交通要道。在公路方面，确保罗阳高速、江罗高速年内全线建成通车，汕湛高速清云段、汕湛高速云湛段、高恩高速、怀阳高速郁南段、云茂高速罗定至信宜段全面动工或加快建设；广梧高速公路思劳立交力争建成使用；推动罗定至德庆高速公路北段提前建设；加快谋划怀阳高速公路郁南至阳江西和广三高速扩建；争取新增约800公里省道升级国道、县道升级省道纳入改造计划；建设农村公路350公里。在铁路机场方面，争取罗岑铁路尽快复工，加快推进湛江-肇庆-广州-河源-梅州（云浮段）的铁路客运专线通道项目前期工作；全面盘活罗定机场资产；大力推动珠三角新干线机场项目选址工作。在水路方面，加快西江航道3000吨级扩能升级改造以及云浮精密机械和海洋装备码头、南江口码头升级建设，积极推动西江大桥项目选址和立项等工作。在城乡公共交通方面，优化城乡一体化大公交网络，积极推广使用氢能源电池公交车等新能源汽车，发展环保低碳公交。大力发展快速公共汽车，不断完善交通调度中心、首末站、停靠站、公共停车场、换乘枢纽、充电桩、充电站等配套服务设施。

（二）加快云浮新区建设。争取新区的地方一般公共预算收入增长10%，规模以上工业总产值增长20%，固定资产投资增长15%。抓好一批重点基础设施，加快规划建设地下管线、排水防涝、供水供电、污水处理、绿地系统、通信等基础性工程。推动新城快线二期、东部快线、南广高铁综合交通枢纽、强盛路东段、站南路网、北湖启动区、西江新城绿化景观工程、东山森林公园等项目建设，促进新老城互动。坚持产城融合，力促华润西江发电厂等项目加快建设。加快推进中央商务区宝能、奥园、敏捷、大润发等城市综合体项目，促进人气聚集。加快广东华立城市职业学院综合项目（二期）、华南师范大学附属云浮学校、佛山中医院云浮分院等公共服务设施的配套规划建设，完善城市功能。积极改善新区群众的生产生活条件，切实解决征地拆迁安置、留用地、失地农民就业、入学等问题。

（三）推进现代城市建设。**加快完善城市基础设施，提升城市功能。**切实抓好城市地下综合管廊规划。继续实施中心城区道路贯通工程，推进东方路、浩林西路、育才路等3条城市道路的贯通。加快城乡结合部的路灯建设。加大城农网改造力度。完成市城区迳尾水库应急水源引水工程。加强城市管道燃气管理，加快建成城区车用燃气加气站，保证管道燃气运行有序。继续推进城市生活垃圾无害化处理，逐步推行生活垃圾分类收集。加快建设各县（市、区）生活垃圾无害化处理场，促进生活垃圾处理减量化、资源化。稳定房地产市场，消化商品房库存。**加强生态环境建设，提升城市品位。**研究出台建设现代生态城市行动纲要。加快开展新型城镇化“2511”综合试点工作。全力推进14个“三旧”改造项目，改造面积327.26亩。科学规划城区市政生态景观项目，增建或扩建一批公共广场和城市景观。巩固创卫创文成果，深入推进“国家园林城市”创建活动。加快数字城管系统建设，提高城市管理水平。

（四）优化区域生态环境。严守生态红线，扎实推进生态保护，实现森林资源增加森林面积、增加森林蓄积“双增”目标。深入实施生态景观林、森林碳汇林、森林进城围城和乡村绿化美化重点生态工程。加强森林资源保护，加快“三江五道”生态走廊建设。尽早消灭全市现有的1万亩宜林荒山，改造2.5万亩疏残林、纯松林和布局不合理的速生林，推进4.2万亩新老城区林相改造。加强环境保护，继续强力实施“五治”工作，大力治理违法违规企业、西江岸线码头、中小河流、车辆超限超载、农村生活垃圾和污水，全面开展圩镇集市垃圾污水治理。强化约束性指标管理，加强高能耗行业能耗管控。严格环评审批监管，所有新上新建项目必须执行环评“三同时”制度。继续深入推进生态文明示范镇、幸福村居、“三个宜居”建设。

三、更加注重民生保障，着力推动惠民利民工程

继续办好十件民生实事，着力解决一批热点难点问题，提高人民群众的获得感和幸福感。

（一）提升就业和社会保障水平。深入实施就业优先战略，不断完善社会保险制度，创新人才人事工作和劳动监察执法机制。**一是促进创业就业。**落实激励创业的各项政策，发挥好基层公共就业服务平台作用，促进大众创业。落实积极的就业政策，探索建立经济发展和扩大就业更加紧密的联动机制，进一步创造就业机会，新增城镇就业2万人。大力开展职业技能培训，实施农村劳动力、被征地农民、新一轮扶贫开发等专项培训工程。**二是促进社会保障全覆盖。**扩大社会保险覆盖面，推动企业全员足额参保，引导城乡居民参保续保，推进机关事业单位工作人员养老保险制度改革。加强城乡低保、特困人员供养、社会福利、养老等工作，每个县（市、区）建设1所省二级以上福利机构和1个城市社区居家养老示范点。加快建立各种社会保障衔接和统一的社会救助体系，健全托底性的社会保障制度。积极推进保障性安居工程建设，开展农村危房改造 4683户，开工建设各类保障性安居工程2612套，基本建成988套。探索把农业转移人口和外来务工人员纳入住房保障范围，解决新就业群体阶段性居住问题。**三是深入开展扶贫开发工作。**坚持精准扶贫、精准脱贫，做实建档立卡、动态管理。采取金融、产业、智力、保障、光伏、电商等扶贫模式，务必实现1.86万户7.03万人有劳动能力相对贫困人口、102个相对贫困村脱贫。

（二）优化公共服务体系。**一是推进公共教育体系建设。**巩固教育创强成果，促进基础教育均衡发展。全面启动 “广东省推进教育现代化先进市”创建工作，加快建成市级智慧教育云平台。推进优秀传统文化进校园。深入实施强师工程，加强教师队伍建设，倡导尊师重教，维护教师权益。继续加大教育投入，完善家庭经济困难学生资助体系，促进教育公平。加快推进开放大学建设。大力发展民办教育、职业教育、高等教育。引导学前教育规范有序发展，重视老年教育和社区教育。**二是推进公共卫生和基本医疗服务体系建设。**巩固扩大基本医疗保障覆盖面，建立健全重大疾病医疗保险和疾病应急救助制度，完善城乡居民大病保险制度。完善卫生计生服务体系，推进卫生强市、卫生强县（市、区）和健康云浮建设。加快建设市人民医院二期工程、市妇幼保健院改扩建项目。继续推进中医药强市建设。加强人口与计划生育工作，落实全面实施“两孩”政策，完善生育登记服务制度，强化妇幼保健能力。加大对计划生育家庭的扶助力度，解决好计划生育特殊家庭保障等问题。**三是推进现代公共文化服务体系建设。**加快建设广东禅文化创意产业园、广东石文化产业园和云浮兰寨南江文化创意产业基地，深化禅宗文化、石艺文化和南江文化与旅游产业的融合发展。完善市、县、镇、村四级公共文化设施网络，发挥好综合文化站、文化室的功能，打造优秀传统特色文化品牌。做好自然村历史人文普查工作。推动历史文化资源保护和开发利用，建设一批非物质文化遗产传承基地。举办云浮市第五届运动会和第三届“市长杯”足球联赛，推动全民健身运动深入开展。

（三）深化社会治理创新。加快基层网格化社会管理的构建与实施，注重为基层群众做好服务，最大限度增加和谐因素。坚持实施“平安云浮”工程，加强“平安细胞”工程建设，构建社会治安立体化防控体系。突出“预防为主，综合防治，禁种、禁制、禁贩、禁吸并举”工作方针，强力推进禁毒工作，加强戒毒人员的帮扶和监管。全面实行重大决策社会稳定风险评估机制和突发事件应急处置机制。整合资源，打造全方位、多层次的反走私综合治理平台。加强互联网监管，强化对网络舆情的引导和管理。落实“党政同责，一岗双责，齐抓共管，失职追责”要求，加强安全生产和食品药品监督管理，防范重特大安全事故和食品药品安全事故发生。建立健全打假工作责任制，严厉打击制售假冒伪劣产品的违法行为。全面推进以城市住宅小区和农村为重点的协同共治体系建设。落实和深化国防和军队改革各项任务，推动国防动员和后备力量建设。

四、更加注重区域协调发展，着力扩大开放与合作

（一）深入推进与佛山市的交流合作。借助佛山的力量加快全市重点产业园区建设，加大一体化招商力度，通过PPP等建设模式，推动产业园区扩能增效和产业发展。加快战略性新兴产业集聚，培育环保装备、氢能科技、智能制造、生物制药、健康养生等产业。思劳片区要加快中空纤维膜血液透析项目、氢燃料电池客车等项目建设，争取年内有一批项目建成投产。继续实施佛山云浮“教育卫生专业人才千人互派计划”。加快“菜篮子”云浮外延基地等民生项目建设。加强与佛山在金融、会展、旅游、文化等方面的合作。

（二）大力推动“广佛肇清云韶”经济圈建设。共同编制区域协调发展总体规划，论证建立产业合作示范区。加快跨界路网对接，优化跨界公交线路，推动车辆便捷通行。加强界河联合整治，开展大气污染联合防治。加强水资源开发利用、流动人口服务管理、打击跨区域犯罪和处置突发事件的协作。推进通信资费一体化、医疗保险异地即时结算、养老保险无障碍转移，以及劳动监察、劳动仲裁、劳务对接、人才交流等合作。主动承接南沙自贸区的辐射，充分发挥好“无水港”的作用。

（三）加快高铁经济带建设。加快粤桂黔高铁经济带合作试验区广东园云浮分园建设，规划云浮东高铁站区作为云浮分园的核心区，重点发展云计算、商务会展、旅游服务、金融创新、电子商务、新能源汽车等产业；规划云浮分园郁南高铁站拓展区、南江口高铁站拓展区、罗定拓展区和新兴拓展区，重点发展特色旅游、高新电子、现代物流、生物制药、绿色日化、现代农业等产业，空间上形成“一核三主四拓展、一轴一带四射线”的总体布局。

（四）深入推进“珠江——西江经济带”规划实施。加强与珠江流域周边地区的全方位合作，在交通设施、医疗保险、食品药品监管、高铁旅游等多个领域开展交流和对接。加强郁南、梧州的粤桂（郁南-龙圩）生态农业高新科技合作示范区的合作共建，推动年产10万头肉羊养殖场项目加快建设；加强罗定与梧州岑溪的对接，争取启动共建粤桂（罗岑）产业合作示范园区。

五、更加注重深化改革，着力增强科学发展源动力

（一）继续深化农村综合改革。纵深推进全国农村改革试验区建设和国家农产品质量安全市创建工作，创新“公司+现代家庭农场”、“公司+理事会+农户”、“合作社+合作社”经营机制，探索建立龙头企业带动型、社会组织协同型、经营主体联结型等不同类型的现代农业服务体系。探索推进新型城镇化、农村一二三产业融合发展模式，在有条件的县（市、区）开展协调发展课题试点，并争取列入国家发展试点单位。创新金融产品、金融服务、金融惠农，推进政策性农业保险。大力推进农村土地承包经营权确权登记颁证，加强农村集体“三资”管理，治理好农村土地“三乱”行为。

（二）继续推进重点领域体制机制改革。制定实施“去、降、补”行动方案，加快推进供给侧结构性改革。深化社会组织体制改革，构建工青妇枢纽型社会组织体系。完善信访工作机制。深化文化体制改革，构建现代公共文化服务体系。推进事业单位法人治理和信用体系建设。全面实施不动产统一登记制度。推进“一门式”政务改革，拓展完善网上办事大厅功能。加快教育体制和现代职业教育改革，全面提升教育综合实力。深化食品药品、安全生产、就业创业、收入分配改革，构建完备的社会保障和服务体系。深化医药卫生体制改革，扩大公立医院改革范围，完善妇幼保健及医疗服务设施，启动建立分级诊疗制度，巩固完善基本药物制度，全面推进平价医疗服务、社会办医、医师多点执业。深化基层民主机制和司法改革，积极稳妥开展地方立法工作，营造公平、公正、法治的社会良好氛围，促进民主法治建设。

（三）继续深化商事制度改革。开展降低实体经济企业成本行动，降低制度性交易成本。进一步转变政府职能，简政放权，推进清理规范中介服务。深入推进企业信用监管、企业经营住所配套政策改革和工商登记标准化改革。把商事制度改革与网上办事大厅建设有效衔接，加快信用云浮建设，促进市场主体增量提质和产业结构优化升级。

六、更加注重效能提升，着力加强法治政府建设

（一）不断深化政府职能转变。加快行政审批制度改革，压减审批事项，提高行政审批效率。完善各层级、各部门职能配置，规范政府职权目录，推动政府事权规范化，机构、职能、权限、程序、责任法定化、公开化。厘清政府与市场、社会的边界，全面推行政府权责清单制度。创新政府服务管理，更多地运用市场化、社会化、信息化等方式，努力提高办事效率和服务水平。

（二）大力推进法治政府建设。加强对政府立法工作的统筹，严格法规起草和规章制定程序。探索制订行政调解工作办法、行政执法与刑事司法衔接规定，加强民生改善、城市安全运行、生态环境保护等方面制度建设。完善立法后评估机制，建立常态化的法规规章清理机制。深入开展法治宣传教育，落实“谁执法、谁普法”工作机制。促进政府工作人员带头学法守法、依法办事，引导全体市民自觉守法、遇事找法、解决问题靠法。健全公共法律服务体系，完善法律援助制度，保证人民群众及时获得有效的法律帮助。加强重大行政决策咨询，把公众参与、专家论证、风险评估、合法性审查、集体讨论决定作为重大行政决策的程序。规范重大行政决策出台前向人大报告的程序。推行政府法律顾问制度，建立法律顾问队伍。建立重大行政决策绩效评估机制，加强对重大行政决策实施情况跟踪反馈和监督检查。探索决策过错认定标准和责任追究启动机制，落实重大行政决策终身责任追究制度及责任倒查机制。

（三）扎实开展勤政廉政建设。严格执行中央“八项规定”，落实好“三严三实”要求，巩固党的群众路线教育实践活动成果，持之以恒纠正“四风”。落实党风廉政建设责任制，扎实推进惩治和预防腐败体系建设，进一步规范政府采购、招投标和国有资产转让行为。大力推进审计全覆盖，加大对重点领域、重点部门、重点项目的全面监管和全程监督，强化事前防范和事中监控。加强市公共资源交易平台建设。全面推行预算公开，加大“三公”经费公开力度，坚决压缩一切不必要的行政开支，确保“零增长”，树立廉洁、勤政、务实、节俭的政府形象。

各位代表！新常态带来新机遇，新定位开启新征程。我们深感使命光荣和责任重大，让我们在中共云浮市委的坚强领导下，齐心协力，开拓进取，扎实工作，向着全面建设现代生态城市、全面建成小康社会的目标大步迈进！
